# Modification of H1N1 Influenza Luciferase Reporter Viruses Using StopGo Translation and/or Mouse-Adapted Mutations

**DOI:** 10.3390/v17091211

**Published:** 2025-09-05

**Authors:** Po-Ling Chen, Guohua Yang, Chet Ojha, Balaji Banoth, Charles J. Russell

**Affiliations:** Department of Host-Microbe Interactions, St. Jude Children’s Research Hospital, Memphis, TN 38105, USA; po-ling.chen@stjude.org (P.-L.C.); guohua.yang@stjude.org (G.Y.); chet.ojha@seattlechildrens.org (C.O.); banoth.balaji@microcrispr.com (B.B.)

**Keywords:** reporter viruses, influenza virus, luciferase, non-invasive imaging

## Abstract

Reporter viruses are valuable tools for studying infections at the cellular level and in living animals. They also enable rapid, high-throughput antiviral drug screening and serological studies. We previously developed a bioluminescence-based reporter virus, rTN09-PA-Nluc, derived from influenza A/Tennessee/1-560/2009 (TN09, pH1N1) in which a NanoLuc (Nluc) reporter protein was fused to the PA protein. Reduced growth of rTN09-PA-Nluc in MDCK cells and mice was restored by mutations arising from mouse adaptation. Here, to test the hypothesis that the growth defect resulted from the PA-Nluc protein fusion, we generated the luciferase reporter virus rTN09-PA-Nluc/SG, which undergoes StopGo translation to yield separate PA and NLuc proteins along with a proportion of the PA-Nluc fusion. The rTN09-PA-Nluc/SG virus had greater protein expression and increased replication in MDCK cells compared to rTN09-PA-Nluc. The reporter virus encoding StopGo translation was superior to the virus without it in bioluminescence-based virus neutralization assays in vitro, providing results in 24 h as opposed to 3 days using unmodified influenza virus and standard neutralization assay protocols. However, the reporter virus encoding StopGo translation remained attenuated in mice. Mouse-adaptive mutations were needed for full virulence and efficient non-invasive imaging in mice. Overall, these findings demonstrate the benefit of incorporating StopGo translation into influenza reporter viruses for in vitro assays, yet mouse-adapted mutations appeared superior in mice.

## 1. Introduction

Influenza A viruses cause annual epidemics, leading to millions of hospitalizations and an estimated 300,000 to 650,000 deaths worldwide each year [1,2]. These viruses pose ongoing challenges to basic research and clinical practice, highlighting the need to improve methods to study virus–host interactions.

Fluorescent and bioluminescent influenza reporter viruses have been developed to study virus infection at the cellular and tissue levels and to screen novel antiviral agents [3,4,5]. Fluorescent reporter proteins are typically used to track viral infection at the cellular and tissue levels [6,7,8] and to study viral ribonucleoprotein (vRNP) trafficking and host proteins involved in infection [9,10]. Bioluminescent reporter proteins such as Gaussia luciferase (GLuc) and NanoLuc (Nluc) are often used to non-invasively monitor virus infection in living animals [11,12,13,14,15].

Belonging to the family *Orthomyxoviridae*, influenza A viruses have 8 gene segments [16]. Packaging signals at both ends of these segments (and within the distal ends of the coding sequences) are critical for incorporation into viral particles and for viral replication. Therefore, packaging signals must be retained in the gene segment carrying a reporter gene [17].

One approach to generating an influenza reporter virus involves inserting a reporter gene either before or after an influenza gene [11,12,13,14,15,18,19,20] (Figure S1B,C). This method often inserts between the reporter and influenza protein a foot-and-mouth disease virus (FMDV) 2A-like peptide (F2A), which facilitates StopGo translation (also called “Stop-Carry On”, ribosome “skipping”, or 2A-mediated “cleavage”). StopGo translation allows the reporter protein and adjacent viral protein to be expressed from the same open reading frame (ORF), yielding three products: the individual reporter protein, the individual influenza protein, and the read-through chimeric protein [21,22]. In contrast to StopGo, a linker between the viral and reporter protein can be used to generate a single fused protein [10,23]. Another strategy to generate an influenza reporter virus involves rearranging the NS segment [6,7,19,24,25,26,27,28,29] (Figure S1D). Additionally, a reporter gene can be inserted as an intron within the sixth segment (neuraminidase, NA) [30] (Figure S1E).

Previously, we characterized an Nluc reporter virus based on A/TN/1-560/2009 (H1N1) called rTN09-PA-Nluc [14]. In this virus, the Nluc reporter protein was fused to the C-terminus of the PA protein by deleting Glycine^18^ and Proline^19^ (based on Donnelly’s numbering [21]) from the 2A linker peptide and Methionine^1^ from Nluc (Figure S2). The PA and PB2 proteins are subunits of the influenza virus RNA-dependent RNA polymerase complex (along with PB1) that are involved in mRNA cap-snatching, viral RNA transcription, and replication. PB2 binds to the 5′ cap of host mRNA, and PA has endonuclease activity. Construction of a linked reporter was done because previous studies using the WSN influenza virus strain had shown that PA could tolerate a linked reporter on its C-terminal end [10,31]. However, rTN09-PA-Nluc was attenuated in MDCK cells and mice [14]. Most reporter viruses are attenuated in vivo and require mutations to restore viral fitness [14,32,33]. In our previous study [14], we mouse-adapted rTN09-PA-Nluc to enhance virus replication and bioluminescence in vitro and in vivo.

Here, we hypothesized that rTN09-PA-Nluc was attenuated, at least in part, due to the fused-protein design. To test this hypothesis, we generated rTN09-PA-Nluc/SG, in which we added to the PA-Nluc linker peptide the necessary Glycine^18^ (2A), Proline^19^ (2A), and Methionine^1^ (NLuc) residues to create a StopGo sequence between PA and Nluc (Figure S2). The rTN09-PA-Nluc/SG reporter virus maintained a similar genome size compared to rTN09-PA-Nluc but resulted in expression of the PA and NLuc proteins separately along with a proportion of the fusion protein. Both reporter viruses and analogous versions containing mouse-adapted mutations were then compared in vitro and in mice.

## 2. Materials and Methods

### 2.1. Ethics Statement

All animal studies were performed following the “Guide for the Care and Use of Laboratory Animals” published by the National Research Council of the National Academies of the United States and approved by the Animal Care and Use Committee of St. Jude Children’s Research Hospital (protocol number 464).

### 2.2. Cells, Media, and Viruses

MDCK (CCL-34), Vero (CCL-81), and LA-4 murine lung adenoma (CCL-196) cells were obtained from the ATCC and cultured at 37 °C, 5% CO_2_. Minimum Essential Medium (MEM, Gibco^TM^, New York, NY, USA) with 5% FBS was used for MDCK cells, Medium 199 (M199, Gibco^TM^) with 5% FBS was used for Vero cells, and Kaighn’s Modification of Ham’s F-12 with L-glutamine (F-12K, ATCC, Manassas, VA, USA) with 15% FBS was used for LA-4 cells. TN09 reporter viruses used in this work are listed in Table 1. For virus amplification, MDCK cells were seeded in a T175 flask one day before infection. The cells were washed twice with 1× dPBS (with Mg^2+^ and Ca^2+^) and inoculated with viruses for 1 h at 37 °C incubator. After inoculation, viruses were removed by washing, and then the cells were overlaid with infection medium (MEM + 4% BSA + 1 µg/mL TPCK-treated trypsin) before incubation at 37 °C for 3 days.

### 2.3. Plasmids

An eight-plasmid system was used to rescue viruses [34]. The cDNA of each segment of A/Tennessee/560-1/2009 (H1N1), referred to as TN09, was cloned into the reverse genetics plasmid pHW2000 as described previously [14]. Also described previously were the construction of pHW2000 plasmids containing reporter-fused PA (PA-Nluc), PB2 from mouse-adapted clone 9 (MA9), and PA-Nluc-D479N, which was PA-Nluc with mouse-adapted mutation PA-D479N [14]. The previous design of PA-Nluc had the PA and Nluc genes linked by GGC AGC GGC CAG CTG TTG AAT TTT GAC CTT CTT AAG CTT GCG GGA GAC GTC GAG TCC AAC CCC, which translates to GSGQLLNFDLLKLAGDVESNP (Figure 2A). rTN09-PA-Nluc/SG, the StopGo-encoding reporter virus first reported here, co-translates PA and Nluc using a complete FMDV 2A peptide. With a gene design based on PA-Nluc, In-Fusion cloning (In-Fusion^®^ HD cloning kit, TAKARA, Tokyo, Japan) was used to insert the sequence GGA CCT GCG GCC GCA ATG after the linker to create the complete 2A peptide, QLLNFDLLKLAGDVESNPGP (Figure S2). Plasmids for StopGo viruses containing mouse-adapted mutations called rPB2-MA9/SG and rPB2-MA9/PA-D479N/SG were also generated in this study using site-directed mutagenesis (Site-directed mutagenesis kit, Agilent, Santa Clara, CA, USA).

### 2.4. Virus Rescue

The viruses generated for this study are listed in Table 1. Eight expression plasmids (3 µg each) were mixed with suspended Vero cells (5 × 10^6^ cells in 300 µL of RPMI-1640 medium), and then transfection was conducted by electroporation (4 mm cuvette, 220 V with 975 µF, BIO-RAD gene pulser II). After electroporation, the cells were incubated with M199 + 5% FBS medium at 37 °C overnight. The next day, the culture medium was replaced with M199 medium containing 1 µg/mL TPCK-treated trypsin. The cells were incubated at 37 °C for 4 days, supplemented daily with TPCK-treated trypsin. Rescued viruses were amplified in MDCK cells [35].

### 2.5. Virus Titration

Median tissue culture infectious dose (TCID_50_) and plaque assays were used to titrate viruses in MDCK cells. Hemagglutination (HA) assays were used to measure the HA titers of viruses using 0.5% turkey red blood cells (TRBCs) based on the standard WHO protocol [36].

### 2.6. Growth Curves

Virus growth curves used MDCK and LA-4 cells incubated at 37 °C. Cells were seeded in 6-well plates one day before infection (6 × 10^5^ cells/well). The infection medium was MEM + 4% BSA + 1 µg/mL TPCK-treated trypsin. On the day of infection, cells were washed twice with 1× dPBS and then incubated at 37 °C with viruses (3 or 0.001 multiplicity of infection, MOI, diluted with infection medium). Samples were harvested daily and then titrated with MDCK cells.

### 2.7. Luciferase Assays

MDCK cells were seeded in 96-well white plates (3 × 10^4^ cells/well with MEM + 5% FBS medium) a day before infection. The infection medium was MEM + 4% BSA + 1 µg/mL TPCK-treated trypsin. On the day of infection, cells were washed with infection medium and then incubated with viruses (3 MOI) at 37 °C for 1 h. After incubation, the inoculum was replaced with fresh infection medium (100 µL/well), and the cells were incubated at 37 °C. The incubation was stopped at 0, 1, 2, 4, and 6 h post-infection. To measure bioluminescence, the medium was removed, and then 20 µL of substrate (Nano-Glo^®^ Luciferase Assay System, Promega, Madison, WI, USA) was added to each well. After a 3 min incubation, bioluminescence was measured using a Synergy H1 microplate reader (Biotek, Winooski, VT, USA).

### 2.8. Western Blots

MDCK cells were infected (MOI = 1) and incubated at 37 °C for 24 h. The cells were treated with RIPA buffer to obtain the total protein. The proteins were separated by SDS-PAGE (NuPAGE^TM^ 4–12% Bis-Tris Gel, Invitrogen^TM^, Waltham, MA, USA) with 1× MOPS buffer (NuPAGE^TM^ MOPS SDS running buffer, Invitrogen^TM^). The proteins were then transferred to PVDF membranes (PVDF/filter paper sandwich, 0.2 µm pore size, Invitrogen^TM^) using 1× transfer buffer (NuPAGE^TM^ transfer buffer, Invitrogen^TM^). The influenza PA protein was detected using rabbit anti-H1N1 PA (C-terminal domain) antibody (Genetex, GTX118991). The Nluc protein was detected using mouse anti-NanoLuc^®^ Monoclonal Antibody (Promega, N7000). The 2A peptide was detected using rabbit anti-2A peptide (Sigma-Aldrich, St. Louis, MI, USA, ABS31-I). The beta-actin protein was detected using mouse monoclonal antibody (SANTA CRUZ BIOTECHNOLOGY, Dallas, TX, USA, sc-47778). The secondary antibodies used in this study were anti-rabbit IgG-HRP (Cell Signal Technology, Danvers, MA, USA, 7074) and anti-mouse IgG-HRP (Cell Signal Technology, 7076S). The substrate was SuperSignal^TM^ West Pico PLUS Chemiluminescent Substrate (Thermo Scientific^TM^, Waltham, MA, USA).

### 2.9. Mouse Studies

Groups of five female DBA/2 mice were used to observe mortality and weight loss. Mice were infected with 750 PFU (30 µL) of virus through intranasal injection. Body weight was measured daily for 14 days. Mice exceeding 25% weight loss were euthanized.

For in vivo imaging, chest hair was removed from DBA/2 mice, and the animals were infected with 750 PFU of virus through intranasal injection. On the day of image acquisition, mice were sedated with isoflurane and given 100 µL of 20-fold diluted substrate (Nano-Glo^®^ Luciferase Assay System, Promega) through retro-orbital injection. Images were captured using a Xenogen IVIS charge-coupled-device (CCD) camera system (Caliper Life Science, Hopkinton, MA, USA) and analyzed with Living Image 4.5 software (Caliper Life Sciences). After image collection, mice were euthanized, and lungs were collected for virus titration.

### 2.10. Neutralization Assays

MDCK cells were seeded in clear (standard neutralization assays) or white (bioluminescence neutralization assays) 96 well plates (3 × 10^4^ cells/well) one day before infection and then washed with 1× dPBS twice before use. Tested sera were treated with 1× receptor-destroying enzyme (RDE) at 37 °C overnight and then inactivated at 56 °C for 30 min. RDE-treated serum was 2-fold serial-diluted with infection medium (MEM + 4% BSA + 1 µg/mL TPCK-treated trypsin). For neutralization, the diluted sera were incubated with viruses (WT or reporter viruses, 100 TCID_50_/well) at 37 °C for 1 h. After neutralization, the washed cells were inoculated with neutralized viruses at 37 °C for 1 h. The inoculum was removed after a one-hour incubation. The cells were covered with infection medium and incubated at 37 °C for 1 or 3 days.

For read-out by bioluminescence, after one day of incubation, medium was removed from the white plates, and 20 µL of substrate (Promega, Nano-Glo^®^ Luciferase Assay System) was added to each well. After 3 min of incubation, bioluminescence was measured using a Synergy H1 microplate reader (Biotek). The neutralization titer (50% inhibition) was calculated by GraphPad Prism 10 using a nonlinear fit with a sigmoidal 4PL curve.

For read-out by HA assay, after three days of incubation, supernatant was removed from clear plates, transferred to 96-well V-bottom plates, and mixed with an equal volume of 0.5% TRBCs. After 40 min incubation at room temperature, the neutralization titer (NT titer) was recorded as the highest dilution leading to 50% infection based on hemagglutination of each well.

### 2.11. Statistical Analyses

All data was analyzed with GraphPad Prism 10 software. *p* values < 0.05 were considered statistically significant. Protein expression levels were compared using ordinary one-way ANOVA followed by Tukey’s multiple comparison test. In vitro virus growth curves and bioluminescence were compared using ordinary two-way ANOVA followed by Dunnett’s multiple comparison. Peak virus titers in MDCK cells and bioluminescence levels at 6 h after infection were compared using ordinary one-way ANOVA followed by Tukey’s multiple comparison test. Viral loads in mouse lungs were compared by ordinary two-way ANOVA, followed by Dunnett’s multiple comparison test. The levels of bioluminescence in mouse lungs were compared by ordinary two-way ANOVA followed by Šidák’s multiple comparison test.

## 3. Results

### 3.1. In Vitro Protein Expression

Seven viruses were studied (Table 1). The three viruses lacking mouse-adapted mutations were rTN-09-WT, rTN09-PA-Nluc (fused reporter gene; abbreviated f_PA-Nluc), and rTN-09-PA-Nluc/SG (StopGo translation; abbreviated sg_PA-Nluc). The two reporter viruses containing mouse-adapted mutations in PB2 were rPB2-MA9 (fused reporter; f_MA9) and rPB2-MA9/SG (StopGo translation; sg_MA9). These viruses had three nucleotide substitutions in PB2 (A473G, C1161T, and C1977T), resulting in the single amino acid mutation PB2-E158G. The final two reporter viruses also contained PA-D479N in addition to PB2-MA9: rPB2-MA9/PA-D479N (fused reporter; f_MA9_479) and rPB2-MA9/PA-D479N/SG (StopGo translation; sg_MA9_479).

**Figure 1:**
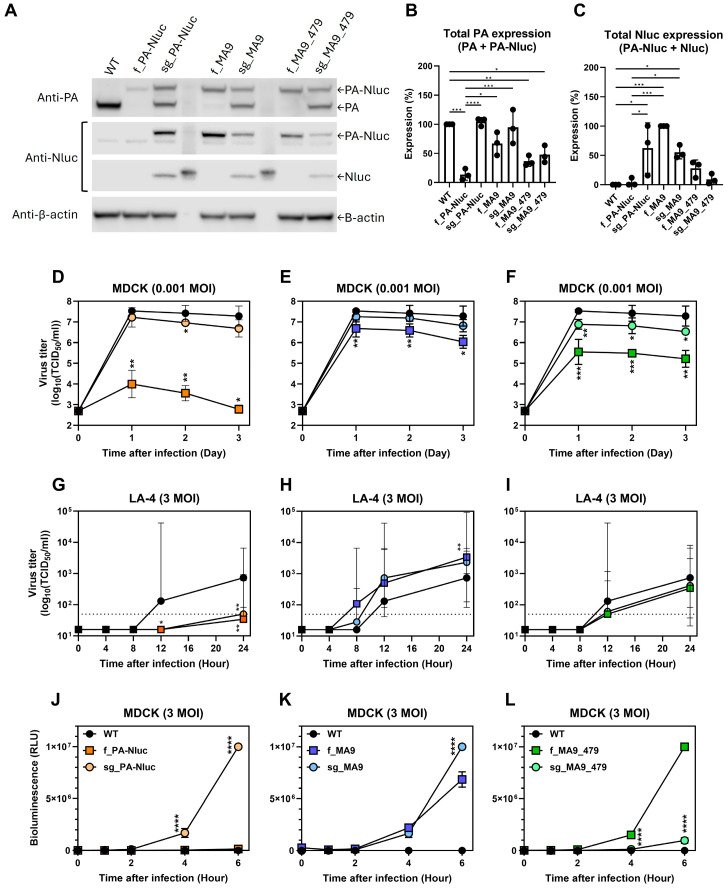
In vitro studies on reporter viruses. (**A**) Expression of PA, Nluc, and PA-Nluc proteins by Western blot (Replicate 2, Figure S3). MDCK cells were infected at an MOI of 1 PFU/cell at 37 °C for 24 h. The Western blot experiment was performed three separate times, and the other two replicates are shown in Figure S3. (**B**) Total expression of PA protein, defined as the sum of fused PA-Nluc and individually expressed PA. The value was normalized to that of the WT virus. (**C**) Total expression of Nluc protein, defined as the sum of fused PA-Nluc plus individually expressed Nluc. The value was normalized to that of f_MA9. For panels (**B**,**C**), the combined data from three biological replicates are shown. Expression between all groups was analyzed by ordinary one-way ANOVA followed by Tukey’s multiple comparisons test. For simplicity, statistically significant differences are only labeled for comparisons to WT and f_PA-Nluc (* *p* < 0.05; ** *p* < 0.01; *** *p* < 0.001; **** *p* < 0.0001). (**D**–**F**) Virus growth in MDCK cells infected at an MOI of 0.001 PFU/cell and incubated at 37 °C. The legends are shown in panels (**J**–**L**). Three biological replicates were performed, and virus growth compared to WT was analyzed by two-way ANOVA followed by Dunnett’s multiple comparisons test (* *p* < 0.05; ** *p* < 0.01; *** *p* < 0.001). (**G**–**I**) Virus growth in murine lung LA-4 cells at an MOI of 3 PFU/cell and incubated at 37 °C. The legends are shown in panels (**J**–**L**). (**J**–**L**) Bioluminescence values of reporter viruses in MDCK cells infected at an MOI of 3 PFU/cell and incubated at 37 °C. Three biological replicates were performed, and to compare fused and StopGo constructs, two-way ANOVA followed by Šidák’s multiple comparisons test was used (**** *p* < 0.0001). Reporter virus pairs were f_PA-Nluc and sg_PA-Nluc (**D**,**G**), f_MA9 and sg_MA9 (**E**,**H**), and f_MA9_479 and sg_MA9_479 (**F**,**I**). The error bars for panels (**B**,**C**,**J**–**L**) are the average with standard variation, while those for panels (**D**–**I**) are the geometric mean with standard variation.

In vitro expression was measured by Western blot in three independent experiments using MDCK-infected cells (Figure 1, Figures S3 and S4). Reporter viruses with the unmodified PA-Nluc gene segment expressed only fused PA-Nluc protein (~100 kDa), while viruses carrying sg_PA-Nluc expressed fused PA-Nluc, PA, and Nluc proteins (Figure 1A). Total PA expression by f_PA-Nluc was only ~13% of WT (*p* < 0.0001). In contrast, sg_PA-Nluc and sg_MA9 had expression similar to WT (*p* > 0.999) (Figure 1B). Total PA expression of f_MA9, f_MA9_479, and sg_MA9_479 was reduced to approximately 67% (*p* = 0.1918), 37% (*p* = 0.0028), and 48% (*p* = 0.0132), respectively. Thus, f_PA-Nluc and the viruses containing PA-D479N had significantly reduced PA expression, whereas sg_PA-Nluc and sg_MA9 had total PA expression like WT. Both StopGo and mouse-adaptive modifications increased luciferase expression compared to f_PA-Nluc (Figure 1C). Notably, f_MA9 had ~24-fold higher luciferase activity than f_PA-Nluc (*p* = 0.0002), and viruses with PA-D479N had relatively low luciferase activity.

### 3.2. StopGo Translation Increased Virus Replication and Bioluminescence in MDCK Cells

Virus growth was measured using MDCK (Figure 1D–F) and LA-4 murine lung adenoma (Figure 1G–I) cells. In MDCK cells infected at an MOI of 0.001 PFU/cell, WT virus had an average peak titer of 4.4 × 10^7^ TCID_50_/mL, whereas f_PA-Nluc was highly attenuated (1.1 × 10^4^ TCID_50_/mL, *p* = 0.001, Figure 1D). The StopGo modification increased the peak titer of sg_PA-Nluc to 1.9 × 10^7^ TCID_50_/mL, which was 1700-fold higher than f_PA-Nluc (*p* < 0.05) but nearly half as much as WT virus (*p* > 0.05). In MDCK cells, sg_MA9 and f_MA9 had peak titers of 2.2 × 10^7^ and 5.8 × 10^6^ TCID_50_/mL, respectively (Figure 1E), and the PA-D479N mutation did not further enhance virus growth (Figure 1F). Virus growth was relatively low in LA-4 murine lung cells infected at an MOI of 0.001 PFU/cell, so subsequent infection experiments were conducted with an MOI of 3 PFU/cell. In LA-4 cells, the WT virus grew to an average titer of approximately 1000 TCID_50_/mL after 24 h of infection, whereas the viruses f_PA-Nluc and sg_PA-Nluc only reached a titer near the limit of detection (Figure 1G). The mouse-adapted MA9 mutations increased reporter virus growth to a level similar to or exceeding WT (Figure 1H,I). In general, the largest effect when comparing virus growth in MDCK vs. LA-4 cells was that the f_PA-Nluc and sg_PA-Nluc viruses lacking mouse-adapted mutations were more highly attenuated in the murine lung cells.

In vitro bioluminescence was measured in MDCK cells (Figure 1J–L). At 6 hpi, sg_PA-Nluc had a bioluminescence level of approximately 1 × 10^7^ RLU, which was 67-fold higher than that of f_PA-Nluc (*p* < 0.0001, Figure 1J). The MA9-containing viruses also had higher bioluminescence (Figure 1K). Because PA-D479N did not enhance virus growth or bioluminescence in vitro, f_MA9_479 and sg_MA9_479 were not studied in mice.

### 3.3. Use of Reporter Viruses in Microneutralization Assays

**Figure 2:**
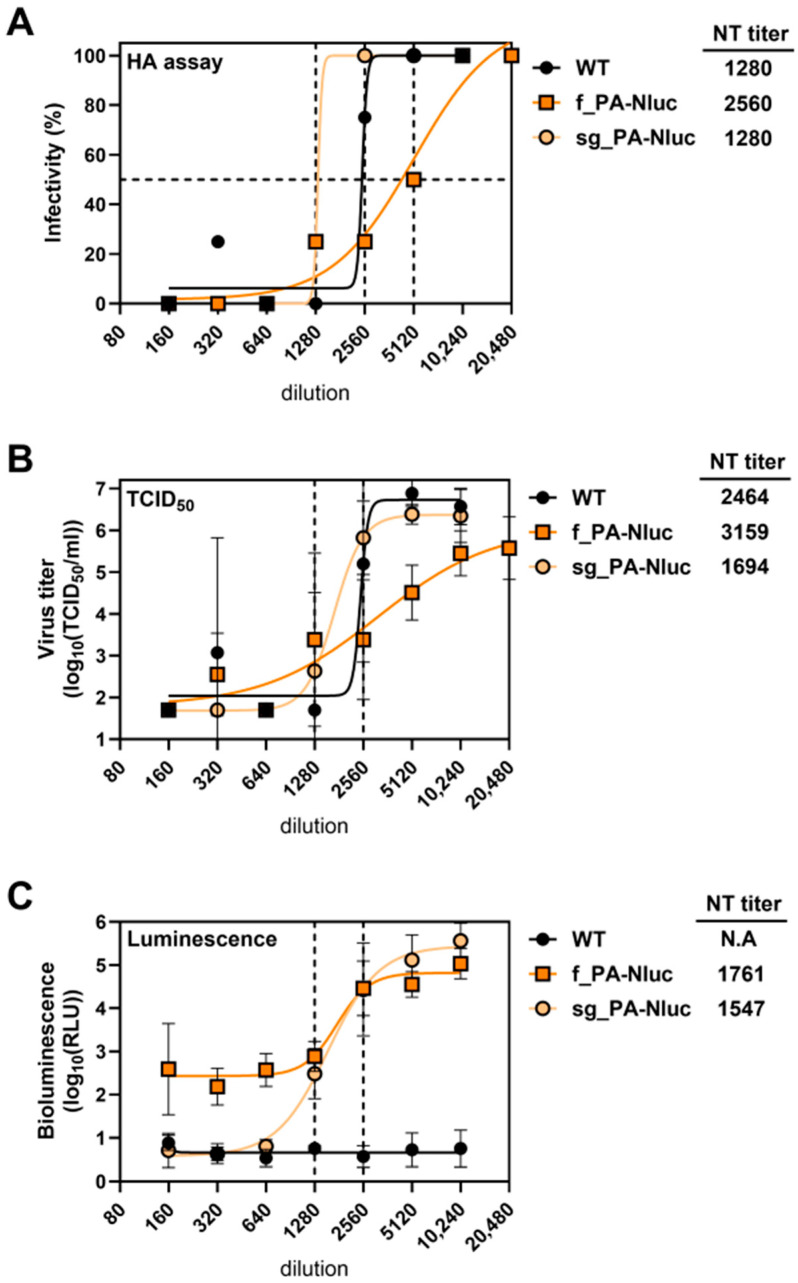
Neutralization assays using reporter viruses. WT and reporter viruses were incubated with a serial diluted mouse anti-TN09 serum before addition to MDCK cells. (**A**) Standard influenza virus neutralization assay. After 3 days of incubation at 37 °C, each well was measured for residual infectivity using the standard hemagglutination assay. The highest dilution resulting in 50% infectivity was defined as the neutralization titer. Values are listed to the right of the panel. (**B**) Neutralization titers measured by TCID_50_ assays. After 3 days of incubation at 37 °C, each well was measured for residual infectivity by TCID_50_ assays in MDCK cells. (**C**) Neutralization titers measured by bioluminescence. After 22 h incubation at 37 °C, residual infectivity was measured by luciferase assay. The experiment was repeated once, and representative data is shown. For all panels, each point represents the average of four technical repeat values. For panels (**B**,**C**), the neutralization titers were calculated using a sigmoidal 4PL curve function using GraphPad Prism, and the error bars are the geometric mean with standard variation.

Standard microneutralization assays for influenza viruses require incubating a mixture of virus and diluted sera, infecting cells, and after three days performing HA assays to measure the dilution of sera that no longer prevents infection. We wished to investigate whether bioluminescence could be used to shorten the incubation time to one day (Table S1). The viruses WT, f_PA-Nluc, or sg_PA-Nluc were treated with serial dilutions of mouse anti-TN09 serum and inoculated into MDCK cells. The final titers of viruses after mixing with diluted sera were 0.003 TCID_50_/mL in all three assays (HA, TCID_50_, and bioluminescence). After 3 days (HA and TCID_50_ readout) or 1 day (bioluminescence readout), the highest dilution yielding 50% inhibition (the neutralization titer) was measured by all three assays (Figure 2). The neutralization titer of WT was 1280 by both HA and TCID_50_ assays (Figure 2A,B). Typically, TCID50 values are not performed for microneutralization assays. However, they were performed here to correlate the effect of serum dilution on infectious virus titer to compare to the bioluminescence assays. sg_PA-Nluc also had a neutralization titer of 1280 by HA assay, while that of f_PA-Nluc was increased to 2560 (Figure 2A). Using the bioluminescence assay, sg_PA-Nluc and f_PA-Nluc had neutralization titers of 1547 and 1761, respectively, and the bioluminescence signal of sg_PA-Nluc had a broader dynamic range (Figure 2C). Overall, bioluminescence was suitable for neutralization readout, and the StopGo modification increased dynamic range.

### 3.4. StopGo Translation Was Insufficient to Restore Virulence in Mice

**Figure 3:**
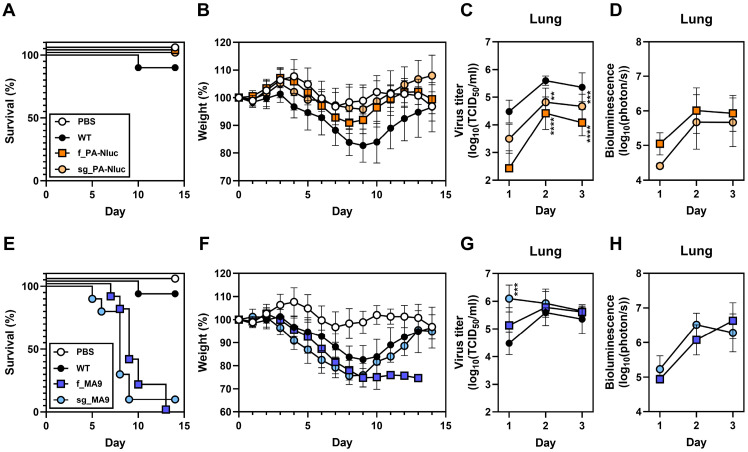
Clinical symptoms of reporter virus infection in mice. DBA/2J mice were intranasally inoculated with 750 PFU of virus. Survival rate (**A**,**E**) and % starting body weight (**B**,**F**) were monitored for 14d after inoculation. (**C**,**G**) Virus loads in lung homogenates collected 1, 2, or 3 days after infection. Statistical analysis compared WT to other groups using two-way ANOVA followed by Dunnett’s multiple comparisons test. (**D**,**H**) Kinetics of lung bioluminescence. Comparisons between fused and StopGo constructs were performed by two-way ANOVA with Šídák’s multiple comparisons test (** *p* < 0.01; *** *p* < 0.001; **** *p* < 0.0001). There were 10 mice/group for weight loss and survival and 5 mice/group for lung titers and bioluminescence. The error bars are the geometric mean with standard variation.

Mouse-adapted mutations were previously shown to restore the virulence of f_PA-Nluc [14]. Here, we investigated the effect of the StopGo modification on virulence. DBA/2J mice were intranasally inoculated with 750 PFU of virus. Mice infected with f_PA-Nluc and sg_PA-Nluc had no mortality and lost less weight than those infected with WT (Figure 3A,B). In contrast, reporter viruses containing the mouse-adapted MA9 mutations, with or without the StopGo modification, were fully virulent (Figure 3E,F).

The effects of StopGo translation and mouse-adapted mutations on in vivo virus growth and bioluminescence were studied after intranasal inoculation of 750 PFU virus into DBA/2J mice. Virus loads from sg_PA-Nluc infection were higher than those from f_PA-Nluc but lower than WT (Figure 3C and Figure S5A). f_MA9 replicated in the lungs like WT, and sg_MA9 grew to higher titers than WT after 1 day of infection (Figure 3G). Thus, mouse-adapted mutations, but not StopGo translation, restored virus growth in vivo.

All four reporter viruses provided sufficient infection for non-invasive imaging (Figure S6) and quantification of bioluminescence (Figure 3D,H). f_PA-Nluc and sg_PA-Nluc had similar bioluminescence kinetics, with f_PA-Nluc trending higher (Figure 3D). For the MA9-containing viruses, bioluminescence was similar regardless of the StopGo modification (Figure 3H). Across all four viruses, peak bioluminescence was similar, although sg_PA-Nluc trended lower than the others (Figure S6E). Overall, the MA9 mutations conferred greater benefit for virulence, virus growth, and bioluminescence in mice than the StopGo modification.

## 4. Discussion

This study investigated the use of StopGo translation and/or mouse-adapted mutations to enhance a 2009 pH1N1 influenza reporter virus called rTN09-PA-Nluc, in which luciferase was appended to the 5′ end of the PA gene [14]. The original rTN09-PA-Nluc virus expressed only the fused PA-Nluc protein from the PA gene segment and was highly attenuated. The rTN09-PA-Nluc/SG virus developed here contained an 18-nucleotide insertion between PA and Nluc that encodes StopGo translation, yielding fused PA-Nluc along with individual PA and Nluc proteins. StopGo translation enhanced infection in MDCK cells by increasing total PA expression, restoring virus growth kinetics to nearly wild-type-like levels, increasing bioluminescence, and enabling 1-day readout of virus neutralization. In contrast, PB2 mouse-adapted MA9 mutations provided superior in vivo effects in mice compared to StopGo by restoring virulence and lung titers to wild-type-like levels. Somewhat disappointing, combining StopGo and mouse adaptations provided little synergy apart from an increase in lung titers in mice after 1 day of infection, but not after 2 or 3 days. Overall, the StopGo strategy was only favorable in a few in vitro applications, while mouse adaptation was superior for in vivo experiments.

Influenza reporter viruses expressing bioluminescent proteins have been successfully used to monitor virus infection in vitro and non-invasively in living mice. This has had many advantages for studies on antiviral therapeutics and vaccines in addition to studying virus dissemination after infection and transmission [11,12,13,14,15,37,38,39]. However, it is often challenging to add nonessential reporter genes to influenza viruses while maintaining viral properties.

The influenza reporter virus rTN09-PA-Nluc, described here and previously [14], inserted a luciferase reporter gene at the 5′ end of the PA gene, resulting in a PA-Nluc fusion protein. This resulted in attenuation. A fused A/WSN/33 H1N1 influenza reporter virus expressing GFP was used to study vRNP trafficking and assembly, and this study showed that a PA protein fusion had little detrimental effect on protein expression and interaction [10]. However, this study did not compare reporter virus growth to that of WT. Other studies with luciferase fused to the C-terminus of PA were also in the background of WSN [15,31]. In A549 cells, growth of rWSN-PA-fused-Nluc was delayed approximately eight hours but reached a peak titer at 24 h, similar to WSN WT [31]. However, subsequent experiments in mice used a StopGo form of rWSN-PA-Stop-Go-Nluc, so it is unknown whether a fused reporter would also be attenuated in the background of WSN. Another study generating a WSN reporter virus with Nluc expressed by StopGo translation after the PB2 protein showed that the virus was useful for drug screening and studying RSV coinfection but did not compare the WSN reporter virus fitness to WT [40]. Here, compared to the reporter virus with StopGo translation, the TN09 reporter virus with PA fused to Nluc was not further attenuated in mice. However, this does not suggest that future Nluc-expressing influenza reporter viruses should use the fused construction, which may only be advantageous when studying protein trafficking using a fluorescent protein.

At least nine manuscripts describe luciferase reporter viruses in the background of the influenza PR8 strain [11,12,20,26,29,30,38,41,42], most of which use the 2A connecting peptide for StopGo translation, but one expresses luciferase from an intron. The reporter genes were inserted before and/or after influenza genes on the PB2, PA, HA, NA, and NS segments. Most of these PR8-based reporter viruses were attenuated in vitro and in vivo despite the use of StopGo translation or an intron to separately express luciferase. The two papers reporting WT-like growth of luciferase-expressing PR8 viruses in MDCK cells and mice used Nluc instead of Gluc, like the other PR8-based studies [30,38]. Therefore, Nluc may be preferred over Gluc. In the present study with PA-Nluc in the background of TN09, the virus was attenuated in mice. In contrast, a PB2-Nluc reporter in the context of the PR8 strain was not attenuating [38]. Further studies are needed to determine the relative contributions of the target gene and virus strain on reporter virus attenuation. Other influenza reporter viruses have been generated in the backgrounds of H3N2, H5N1, H7N7, and H9N2 strains [19,30,39,43,44]. These have also been shown to be useful tools for antiviral and serological screening in addition to studying in vivo infection and transmission.

Various strategies can be used to mitigate influenza virus attenuation due to the addition of nucleic acids and/or the expression of proteins from a foreign reporter gene. For reporter viruses encoding StopGo translation, the sequence of the connecting peptide helps determine the ratio of expression of fused versus individual proteins. The 2A peptide from *Thosea asigna* virus (T2A) has been shown to yield a larger proportion of individual proteins compared to fused than the 2A peptide from porcine teschovirus-1 (PTV 2A) [21]. The 2A peptide from FMDV (F2A) was used here, as it was previously used without attenuation in the context of the A/WSN/33 influenza virus [15]. However, F2A has been shown to yield an even smaller fraction of individual proteins than T2A and PTV 2A [21]. Thus, the T2A peptide may be superior for reporter gene construction. Indeed, a virus containing the 2A peptide from the *Thosea asigna* virus had WT-like fitness and virulence in MDCK cells and mice [38]. Three out of six studies using the PTV 2A peptide reported attenuation in animal models [13,15,19,31,45,46], while the FMDV 2A peptide (F2A) has been shown to result in attenuation in mice [11,14,20]. Thus, optimizing the connecting peptide between genes may be a useful strategy to optimize reporter viruses.

The UTR regions of influenza viruses regulate transcription and replication of vRNAs [47,48]. Spronken et al. introduced two different modifications (2UP and 3UP) into the UTR region of the reporter gene segment, resulting in a reporter virus (2UP_PA_iRFP_dPR) capable of inducing WT-like weight loss and replication in mice [20]. Zhao et al. modified the UTR region to restore the fitness of Gluc-containing PR8 reporter viruses (PR8-NS^CE1^-Gluc) [49]. In this design, the MLD_50_ of PR8-NS^CE1^-Gluc was ~100 TCID_50_ in BALB/c mice, which is close to the MLD_50_ of PR8 (50–100 PFU).

Protein function may be affected by reporter-gene fusion proteins. Reuther et al. found that their original design creating NEP-fused reporter led to a growth deficiency of the SC35M (H7N7) virus in MDCK cells. After introducing an additional 2A peptide to separate the NS1, NEP, and the reporter proteins, in vitro growth of the virus was restored [19]. With respect to rTN09-PA-Nluc (studied previously [14] and here), reduced polymerase activity and virus replication most likely occur due to both lower PA expression and the fusion of PA to Nluc. Both of these are partially overcome by StopGo translation. PB2 residue 158 mutation in the mouse-adapted MA9 variant is located in a region that interacts with PB1 and NP [50,51]. The PB2-E158G mutation in the MA9 variant is thought to promote replication by enhancing formation of the polymerase complex, which includes fused PA-Nluc. Two studies have shown that a PB2-E158G mutation enhances polymerase activity and fitness in mice [52,53].

The classical method to overcome in vivo attenuation of influenza viruses in mice is serial passage and selection of clones with higher replication and/or virulence. Mutants enhancing polymerase activity and receptor binding are often selected during the adaptation. The best-known mutation enhancing influenza polymerase activity in mammals is PB2-E627K [54,55]. PB2-E158G, PB2-D701N, and PA-T97I also increase polymerase activity in vitro and in vivo [53,55,56,57]. HA mutations selected during mouse adaptation enhance binding to sialic acid-containing receptors in the mouse respiratory tract [58,59].

Mouse adaptation has also been used to restore the fitness of reporter viruses. Fukuyama et al. inserted a fluorescent reporter gene in the NS segment (WT-Venus-PR8), resulting in an MLD_50_ more than 100-fold higher than that of the WT virus (>10^4.3^ and 10^2.5^ PFU, respectively). Moreover, the reporter gene was lost after several passages. A mouse-adapted reporter virus (MA-Venus-PR8) containing PB2-E712D and HA-T380A had a lower MLD_50_ (10^3.3^ PFU) and a more stable reporter gene [7]. The PB2-E712D mutation enhanced the transcription/replication efficiency, which in turn increased expression of fused NS1-Venus protein [60].

Several limitations of this study could be addressed to optimize influenza reporter viruses. First, the relative expression of fused versus individual proteins could be compared directly by generating reverse-genetics viruses that differ only in the StopGo amino acid sequence (i.e., T2A-, F2A-, and PTV2A-containing reporter viruses). Second, the effect of reporter gene insertion site could be assessed by positioning Nluc before or after PB2, PA, HA, or NA and by comparing insertions into the NS segment. This should be done at the same time in the same lab and, perhaps, be repeated by another lab. Third, identical Nluc insertions could be introduced into different influenza virus strain backgrounds for direct comparison of strain-specific effects. Finally, the effects of StopGo and mouse-adapted mutations could be studied in other animal models, including ferrets and possibly guinea pigs, to determine whether the findings extend beyond mice.

In summary, a variety of strategies have been used to reduce or overcome attenuation resulting from foreign gene insertion into influenza viruses. Future studies could systematically compare these strategies, individually and in combination, in the backgrounds of varied influenza strains. When designing a new influenza reporter virus, an expedient approach may be to use multiple strategies in parallel, ultimately advancing a candidate based on the most essential phenotype(s) needed for the intended application.

## Figures and Tables

**Table 1:** Viruses in this study.

Virus Name	Abbreviation	Design	PB2 Gene	PA Gene	Reference
rTN09-WT ^1^	WT	-	WT	WT	[14]
rTN09-PA-Nluc	f_PA-Nluc	fused	WT	PA-Nluc ^2^	[14]
rPB2-MA9	f_MA9	fused	A473G/C1161T/C1977T (MA9) ^3^	PA-Nluc	[14]
rPB2-MA9/PA-D479N	f_MA9_479	fused	A473G/C1161T/C1977T (MA9)	PA-Nluc (D479N) ^4^	[14]
rTN09-PA-Nluc/SG	sg_PA-Nluc	StopGo	WT	sg_PA-Nluc ^5^	This study
rPB2-MA9/SG	sg_MA9	StopGo	A473G/C1161T/C1977T (MA9)	sg_PA-Nluc	This study
rPB2-MA9/PA-D479N/SG	sg_MA9_479	StopGo	A473G/C1161T/C1977T (MA9)	sg_PA-Nluc (D479N) ^6^	This study

^1^ rTN09-WT is the reverse-genetics-derived wild-type virus rg-A/Tennessee/1-560/2009 (H1N1) of the pandemic lineage. ^2^ The previous design in which PA and Nluc are translated into a chimeric protein with a 21 amino acid residue linker. ^3^ PB2 gene from mouse-adapted clone 9 (MA9) carrying 3 nucleotide changes (A473G, C1161T and C1977T) resulting in one amino acid mutation (PB2-E158G). ^4^ The PA gene consists of PA-Nluc and encodes for the additional mouse-adapted PA amino acid mutation D479N. ^5^ In sg_PA-Nluc, the PA and Nluc genes are linked by the complete FMDV 2A (F2A) peptide that translates PA and Nluc proteins to enable translation with a StopGo mechanism. ^6^ sg_PA-Nluc with mouse-adapted PA mutation D479N.

## Data Availability

Data available from the authors upon request.

## Supplementary Materials

The following supporting information can be downloaded at: https://www.mdpi.com/article/10.3390/v17091211/s1.

## Abbreviations

The following abbreviations are used in this manuscript:

CCD	charge-coupled device
FBS	fetal bovine serum
FMDV	foot-and-mouth disease virus
GLuc	Gaussia luciferase
HA	hemagglutinin
HRP	horseradish peroxidase
IC50	half-maximal Inhibitory Concentration
IVIS	in vivo imaging system
MA9	mouse-adapted clone 9
MLD50	mouse median lethal dose
MOI	multiplicity of infection
MOPS	3-(N-morpholino) propanesulfonic acid
mPS	modified packaging signal
mSA	modified splicing acceptor
NA	neuraminidase
NEP	nuclear export protein
Nluc	NanoLuc
NP	nucleoprotein
NS1	nonstructural protein 1
NT titer	neutralization titer
ORF	open reading frame
PA	polymerase acidic protein
PB2	polymerase basic 2 protein
PFU	plaque-forming unit
pPS	partial packaging signal
PS	packaging signal
PTV	porcine teschovirus
PVDF	polyvinylidene difluoride
RDE	receptor destroying enzyme
RIPA buffer	radioimmunoprecipitation assay buffer
RLU	relative light units
SA	splicing acceptor
SD	splicing donor
TCID50	median tissue culture infectious dose
TN09	A/Tennessee/1-560/2009
TPCK-treated trypsin	tosyl phenylalanyl chloromethyl ketone-treated trypsin
TRBCs	turkey red blood cells
UTR	untranslated region
vRNA	viral RNA
vRNP	viral ribonucleoprotein
WHO	World Health Organization
WT	wild-type
