# Supplementary material for: Modification of H1N1 Influenza Luciferase Reporter Viruses Using StopGo Translation and/or Mouse-Adapted Mutations
Source: Viruses. 2025 Sep 5;17(9):1211. doi: 10.3390/v17091211 (PMC12474268; doi:10.3390/v17091211)
Supplement: Supplementary file 1 [file viruses-17-01211-s001.zip › viruses-3783024-supplementary.pdf]

Article

Modification of H1N1 influenza luciferase reporter viruses using StopGo translation and/or mouse-adapted mutations

Supplementary data

Table S1. Comparison of neutralization assays using bioluminescence, $TCID_{50}$, and HA assays.

Read out by	Substrate	Incubation time (Days)	Readout time
HA assay	0.5% TRBC	3	40 min
$TCID_{50}$ assay	MDCK cells	3	3 days
Bioluminescence	Luciferin	1	3 min

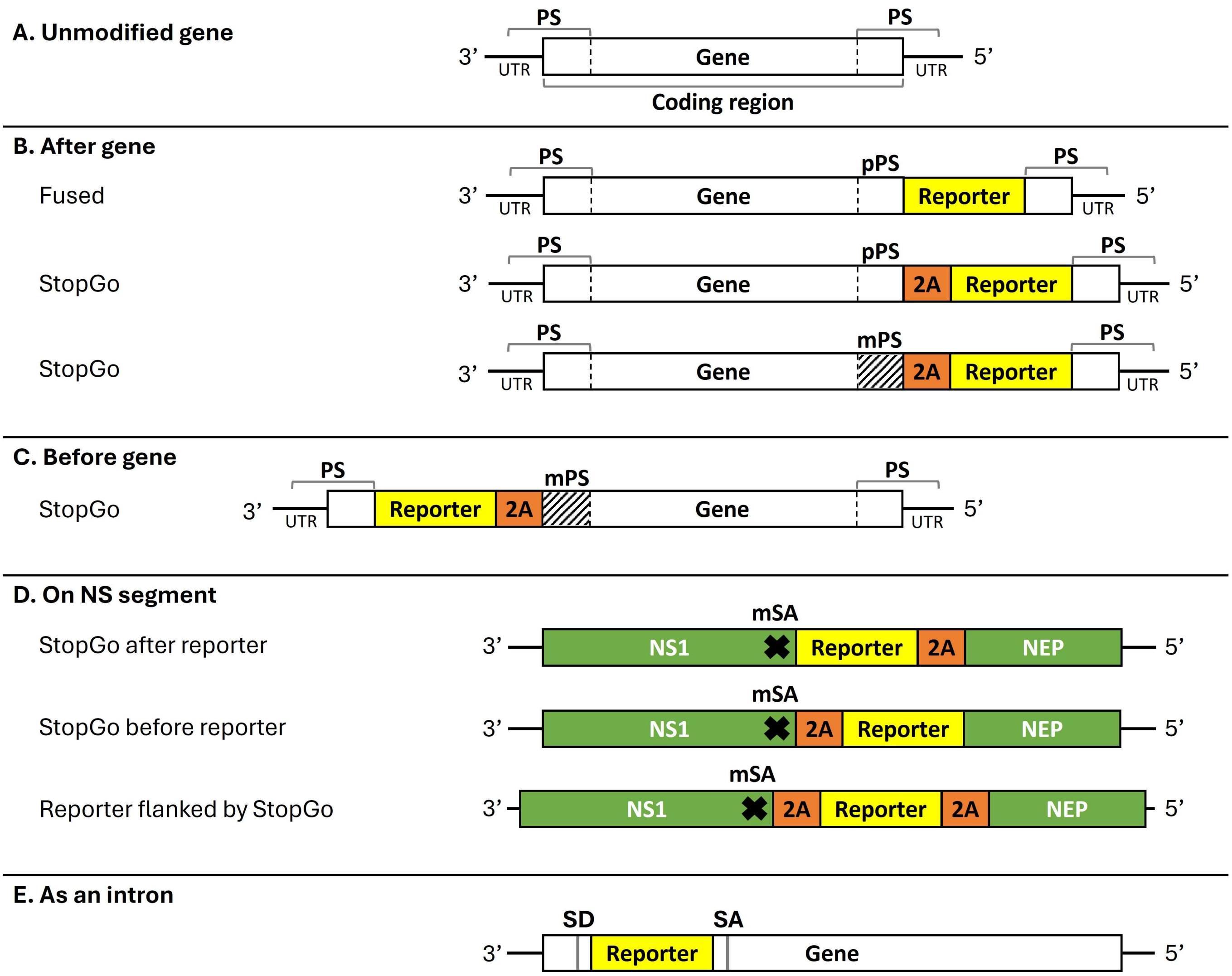

Figure S1. Strategies to incorporate reporter genes into influenza A virus gene segments. (A) Unmodified gene, displayed for reference. (B) Reporter gene added after an influenza virus gene. When encoding StopGo translation, influenza and reporter genes are linked with a 2A sequence. For segment assembly, the original packaging signal on the 5′ end of the target gene is modified with synonymous mutations, and another bona fide packaging signal is added after the reporter gene. Target gene: PA [1-4], PB2 [5], NA [6]. In another design, the influenza and reporter genes are separated by a short linker (e.g., AAA) to create a fused protein. Target gene: PA [7], PB2 [8]. (C) Reporter gene added before the influenza virus gene with the two genes are separated by a 2A sequence. A bona fide packaging signal is added before the reporter gene for virus assembly, and the 3′ end of the packaging signal of target gene is modified. Target gene: hemagglutinin, HA [9], NA [6]. (D) NS segment is rearranged. In this method, the splicing acceptor is modified to avoid splicing. The 2A sequence may be added after or before the reporter gene to create reporter-fused NS1 [9-11] or reporter-fused NEP [12], respectively, or added at both sides of reporter gene to separate NS1, reporter, and NEP proteins [12]. (E) Reporter gene added as an intron. The reporter gene is flanked by a splicing donor and an acceptor. This allows the segment to generate the reporter gene (with a partial N-terminal end of target gene) and the influenza virus gene after splicing. Target gene: NA or NP [13]. pPS: partial packaging signal; mPS: modified packaging signal; 2A: 2A peptide; PS: packaging signal; mSA: modified splicing acceptor; SD: splicing donor; SA: splicing acceptor.

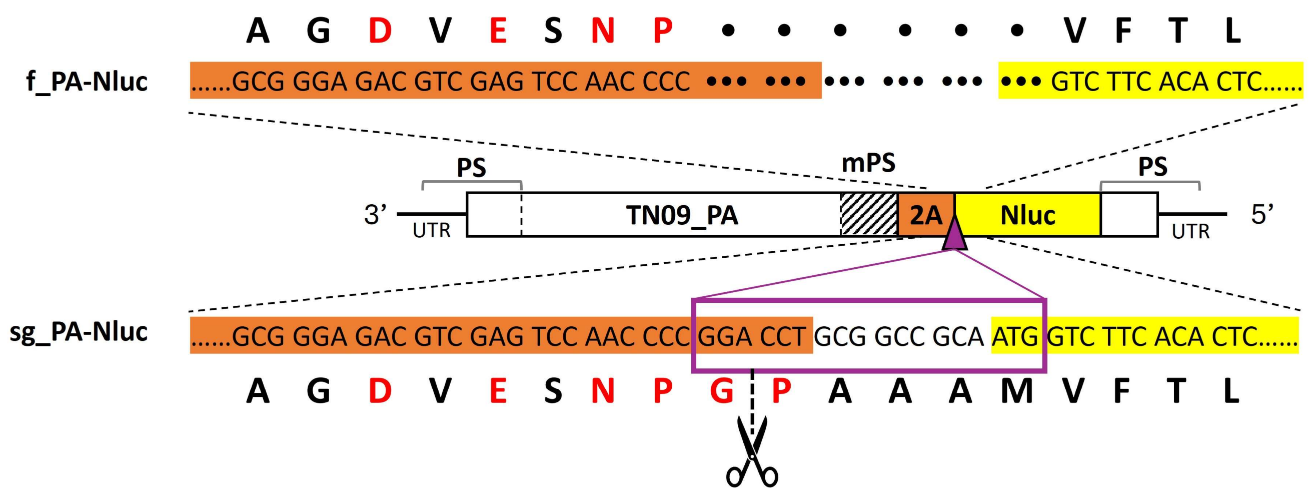

Figure S2. Design of f_PA-Nluc (fusion) and sg_PA-Nluc (StopGo) gene segments. mPS: modified package signal; 2A: FMDV 2A peptide; Nluc: NanoLuc; PS: package signal. Essential amino-acid residues in 2A are highlighted red.

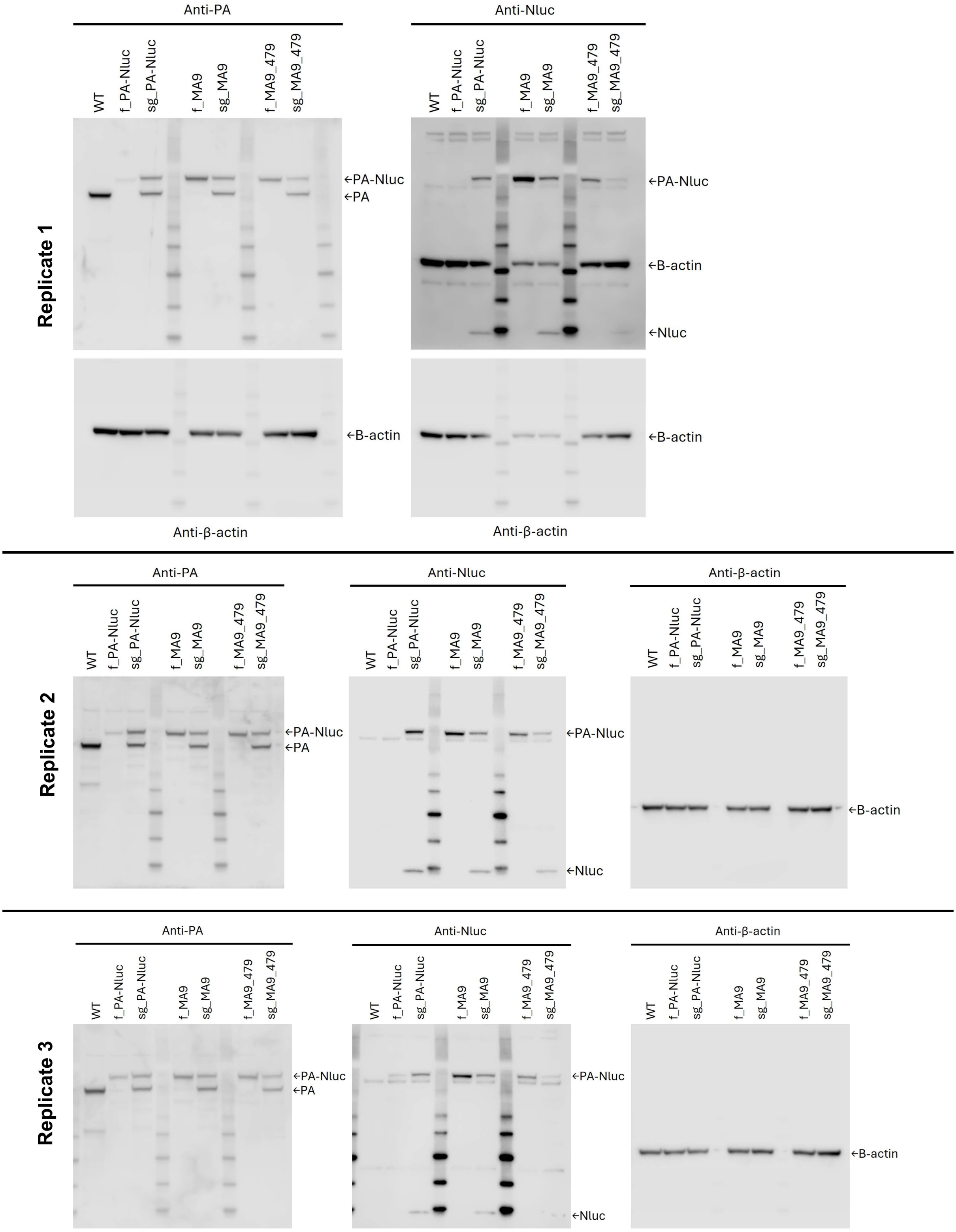

Figure S3. The expression of viral proteins by Western blot. MDCK cells were infected at an MOI of 1 PFU/cell at 37°C for 24h.

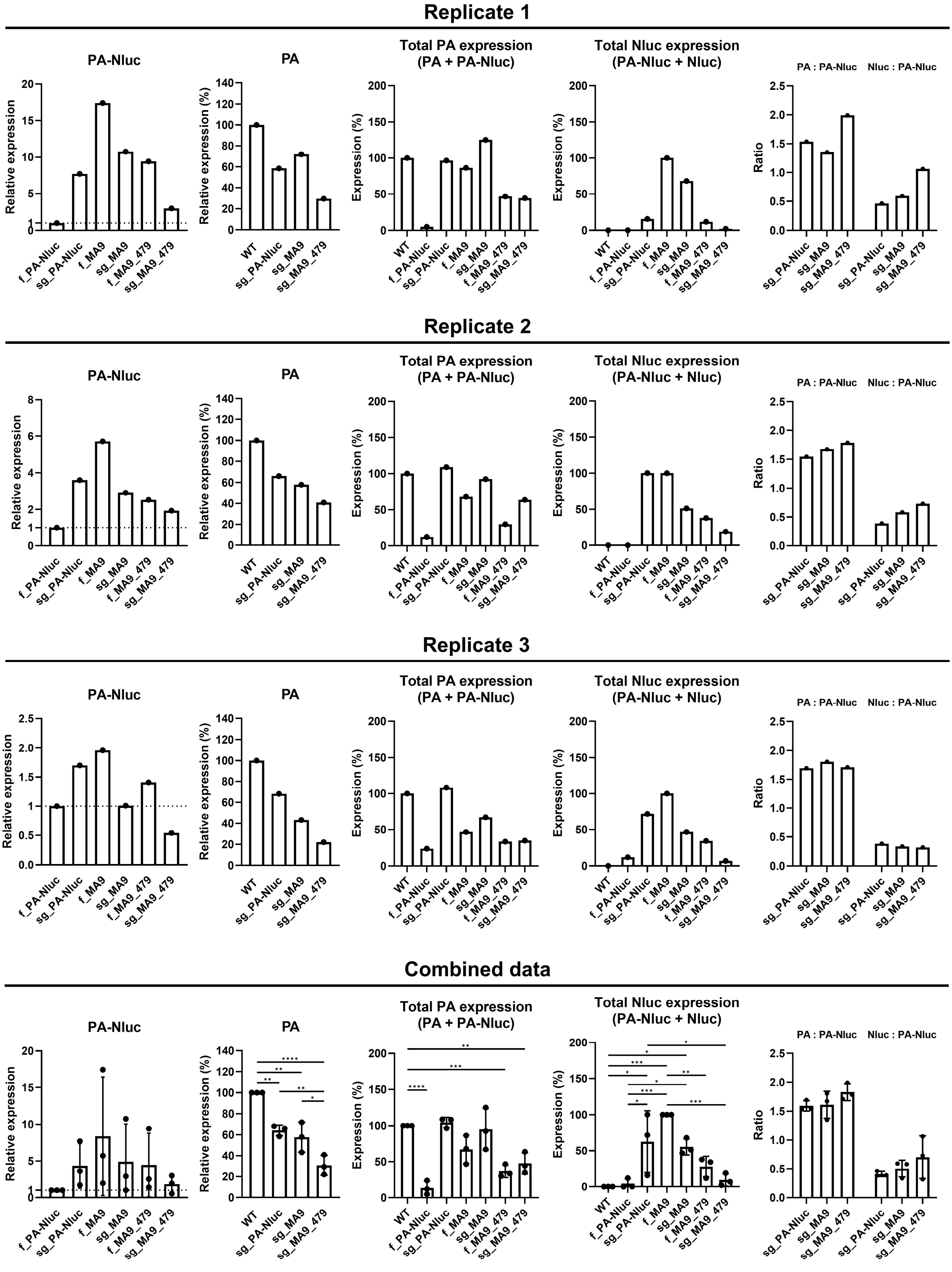

Figure S4. Quantification of viral expression based on the western blot from Figure S3. The bars for the combined data correspond to the average and standard variation.

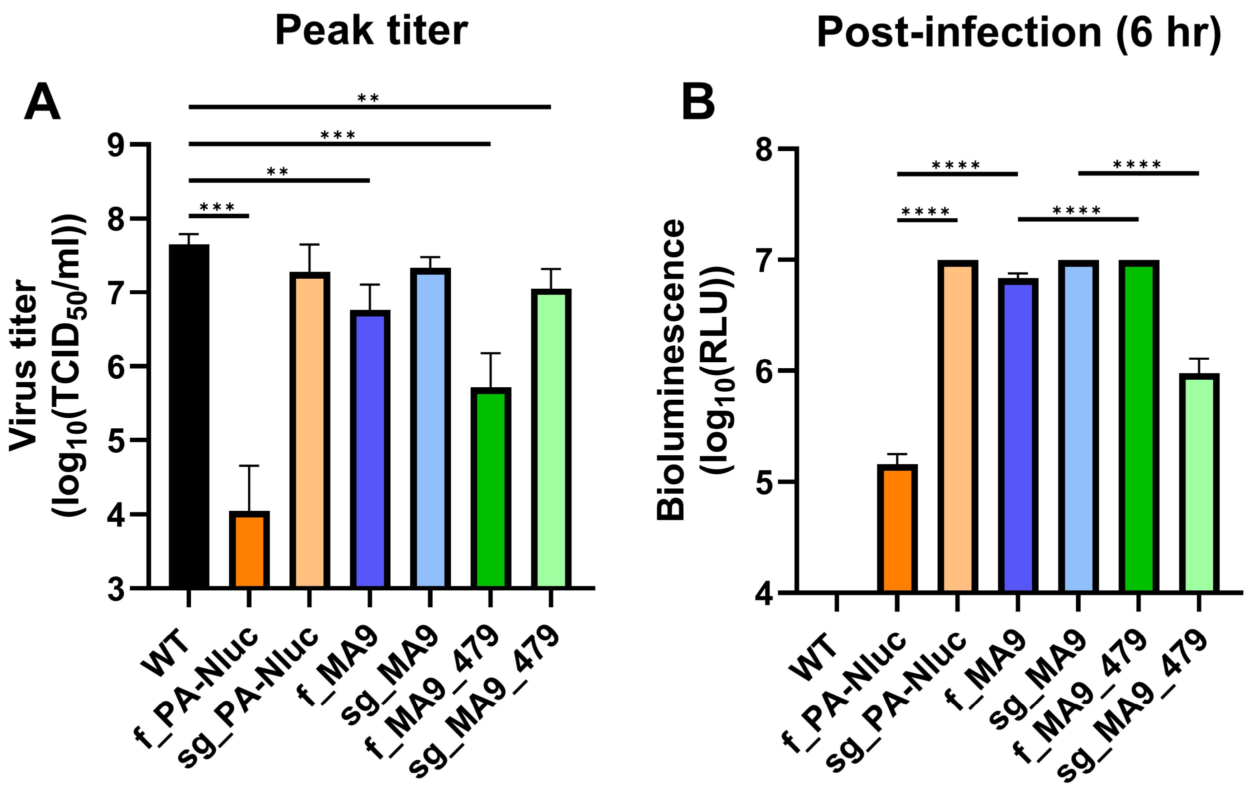

Figure S5. *In vitro* virus growth and bioluminescence of TN09 reporter viruses. (A) the peak titer of the growth of reporter viruses. MDCK cells were infected with 0.001 MOI and incubated at 37°C for 3 days. Samples were collected every day after infection. (B) Bioluminescence at 6 hpi. MDCK cells were infected at an MOI of 3 PFU/cell and incubated at 37°C. The bioluminescence intensity was read at 6 hpi. The bars are the geometric mean with the geometric standard variation. The statistical analysis was based on three biological replicates using ordinary one-way ANOVA with Tukey's multiple comparisons test. (**$p<0.01$; ***$p<0.001$; ****$p<0.0001$).

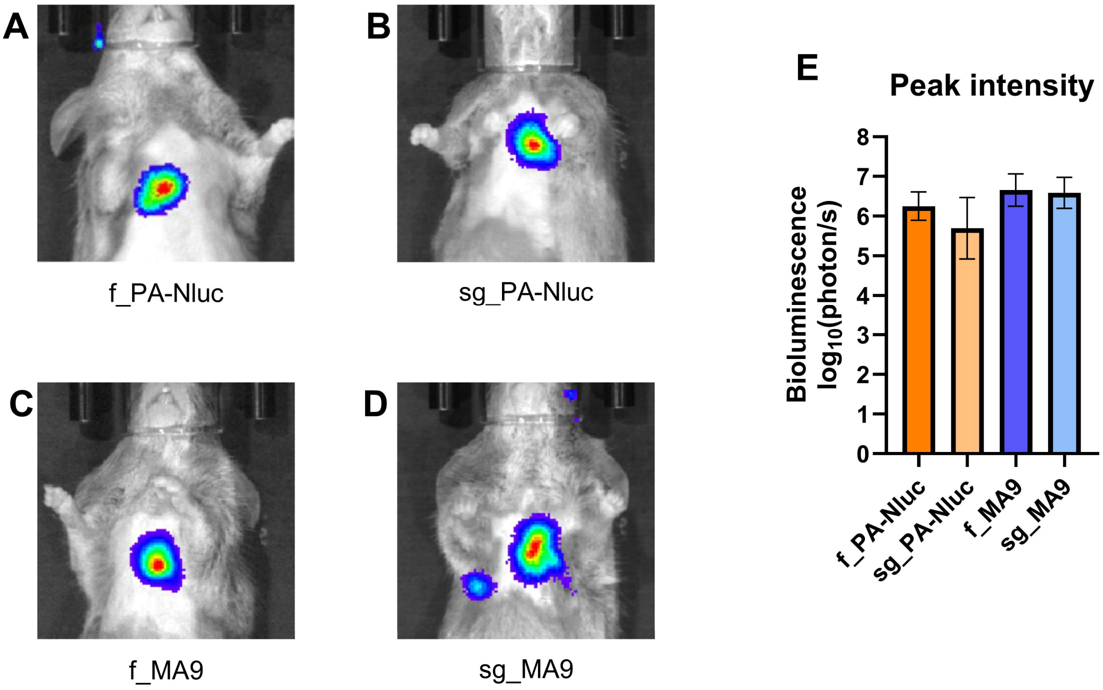

Figure S6. The bioluminescence in mice. (A-D) Representative images of lung bioluminescence emitting from living mice. (E) Peak intensity of lung bioluminescence. Five mice were in each group. The bars in panel E represent the geometric mean with geometric standard variation. Statistical analyses used ordinary one-way ANOVA followed by Tukey's multiple comparisons test. All groups in panel E had $p>0.05$ compared to each other.

Reference

1. Tran, V.; Moser, L.A.; Poole, D.S.; Mehle, A. Highly sensitive real-time in vivo imaging of an influenza reporter virus reveals dynamics of replication and spread. *J Virol* **2013**, *87*, 13321-13329, doi:10.1128/JVI.02381-13.
2. Karlsson, E.A.; Meliopoulos, V.A.; Savage, C.; Livingston, B.; Mehle, A.; Schultz-Cherry, S. Visualizing real-time influenza virus infection, transmission and protection in ferrets. *Nat Commun* **2015**, *6*, 6378, doi:10.1038/ncomms7378.
3. Spronken, M.I.; Short, K.R.; Herfst, S.; Bestebroer, T.M.; Vaes, V.P.; van der Hoeven, B.; Koster, A.J.; Kremers, G.J.; Scott, D.P.; Gultyaev, A.P.; et al. Optimisations and Challenges Involved in the Creation of Various Bioluminescent and Fluorescent Influenza A Virus Strains for In Vitro and In Vivo Applications. *PLoS One* **2015**, *10*, e0133888, doi:10.1371/journal.pone.0133888.
4. Cai, H.; Liu, M.; Russell, C.J. Directed Evolution of an Influenza Reporter Virus To Restore Replication and Virulence and Enhance Noninvasive Bioluminescence Imaging in Mice. *J Virol* **2018**, *92*, doi:10.1128/JVI.00593-18.
5. Heaton, N.S.; Leyva-Grado, V.H.; Tan, G.S.; Eggink, D.; Hai, R.; Palese, P. In vivo bioluminescent imaging of influenza a virus infection and characterization of novel cross-protective monoclonal antibodies. *J Virol* **2013**, *87*, 8272-8281, doi:10.1128/JVI.00969-13.
6. Li, F.; Feng, L.; Pan, W.; Dong, Z.; Li, C.; Sun, C.; Chen, L. Generation of replication-competent recombinant influenza A viruses carrying a reporter gene harbored in the neuraminidase segment. *J Virol* **2010**, *84*, 12075-12081, doi:10.1128/JVI.00046-10.

7. Lakdawala, S.S.; Wu, Y.; Wawrzusin, P.; Kabat, J.; Broadbent, A.J.; Lamirande, E.W.; Fodor, E.; Altan-Bonnet, N.; Shroff, H.; Subbarao, K. Influenza a virus assembly intermediates fuse in the cytoplasm. *PLoS Pathog* **2014**, *10*, e1003971, doi:10.1371/journal.ppat.1003971.
8. Avilov, S.V.; Moisy, D.; Naffakh, N.; Cusack, S. Influenza A virus progeny vRNP trafficking in live infected cells studied with the virus-encoded fluorescently tagged PB2 protein. *Vaccine* **2012**, *30*, 7411-7417, doi:10.1016/j.vaccine.2012.09.077.
9. Nogales, A.; Avila-Perez, G.; Rangel-Moreno, J.; Chiem, K.; DeDiego, M.L.; Martinez-Sobrido, L. A Novel Fluorescent and Bioluminescent Bireporter Influenza A Virus To Evaluate Viral Infections. *J Virol* **2019**, *93*, doi:10.1128/JVI.00032-19.
10. Furusawa, Y.; Yamada, S.; da Silva Lopes, T.J.; Dutta, J.; Khan, Z.; Kriti, D.; van Bakel, H.; Kawaoka, Y. Influenza Virus Polymerase Mutation Stabilizes a Foreign Gene Inserted into the Virus Genome by Enhancing the Transcription/Replication Efficiency of the Modified Segment. *mBio* **2019**, *10*, doi:10.1128/mBio.01794-19.
11. Manicassamy, B.; Manicassamy, S.; Belicha-Villanueva, A.; Pisanelli, G.; Pulendran, B.; Garcia-Sastre, A. Analysis of in vivo dynamics of influenza virus infection in mice using a GFP reporter virus. *Proc Natl Acad Sci U S A* **2010**, *107*, 11531-11536, doi:10.1073/pnas.0914994107.
12. Reuther, P.; Gopfert, K.; Dudek, A.H.; Heiner, M.; Herold, S.; Schwemmle, M. Generation of a variety of stable Influenza A reporter viruses by genetic engineering of the NS gene segment. *Sci Rep* **2015**, *5*, 11346, doi:10.1038/srep11346.
13. Froggatt, H.M.; Burke, K.N.; Chaparian, R.R.; Miranda, H.A.; Zhu, X.; Chambers, B.S.; Heaton, N.S. Influenza A virus segments five and six can harbor artificial introns allowing expanded coding capacity. *PLoS Pathog* **2021**, *17*, e1009951, doi:10.1371/journal.ppat.1009951.
